# Deliberate Self-Harm Among Chinese Children with Different Types and Severity of Disabilities

**DOI:** 10.3390/ijerph16173149

**Published:** 2019-08-29

**Authors:** Huiping Zhu, Xiayidanmu Abudusaimaiti, Joe Xiang, Qi Gao

**Affiliations:** 1 Department of Epidemiology and Health Statistics, School of Public Health, Capital Medical University, Beijing 100069, China; 2 The Center for Intervention Research in Schools, Department of Psychology, College of Arts & Sciences, Ohio University, Athens, OH 45701, USA

**Keywords:** deliberate self-harm, children, disability, Chinese

## Abstract

*Background:* The aims of this study were to determine the prevalence of deliberate self-harm (DSH) and to compare the risk of self-harm in Chinese children with different types and severity of disabilities. *Methods:* Participants were 1300 children aged 6–17 years in Beijing, China; 650 children with disabilities and 650 healthy peers matched on age, gender and residence district. Questionnaires were completed anonymously by parents or children if the age or disability made it necessary. The associations between the disability type, severity and DSH were examined using the Chi-square testing and logistic regression models. *Results:* Children with a single disability or multiple disabilities had statistically higher rates of DSH than children without disabilities (15.6% and 39.7% compared to 10.9%). The prevalence of DSH was associated with the severity of disability, being 36.2% among children with level 1 (the most severe) disability, 19.8% among children with level 2 disability, and 9.2% among children with level 3 + 4 disability. The multivariable odds ratio (OR) of DSH among children with any disability was statistically higher than that among children without a disability (OR = 2.40; 95% CI = 1.71, 3.36; *p* < 0.05). Children with multiple disabilities (OR = 6.89; 95% CI: 4.24–11.20) and level 1 severity of disability (OR = 6.11; 95% CI: 3.91–9.56) had the highest risk of DSH. *Conclusions:* This study clearly demonstrated associations between the severity and type of disability and DSH. This finding highlights the importance of DSH in children with disabilities, and underlines the importance of the prevention of DSH among a vulnerable pediatric population in China.

## 1. Introduction

Deliberate self-harm (DSH) is a major public health problem around the world, with approximately 3% of the total disease burden among people 10–24 years old attributable to self-harm behaviors [1]. The estimated number of individuals who engage in self-injury will be 15–30 million annually by 2020 [2]. Self-harm is typically defined as direct and deliberate bodily harm in the absence of suicidal intent, which is differentiated from suicide attempt, in which there is some intent to die [3]. There is a strong connection between self-harm and suicide, for a history of self-harm is the most important risk factor for suicide [4,5]. One in every 10 children worldwide lives with some form of disability [6,7]. In China, over 5.06 million children aged 17 and under have a disability [8]. Children with disabilities have long-term physical, mental, intellectual or sensory impairments that may reduce their ability to conduct activities and social functions, and hence increases their risk of injury [9,10,11,12]. Due to their varying disability types and functional levels, children with disabilities also have differential injury risks [10,11]. 

Self-harm appeared to be more common amongst individuals with a severe/profound degree of intellectual disability (ID), autism, or deficits in receptive and/or expressive communication [13]. Other studies have found a relation between poor self-image and DSH [14,15]. In addition, children who self-harm tend to have poor insight and coping skills, report feeling significantly more anxiety and hostility, or have a very limited ability to verbally express their feelings [15]. Whilst there have been numerous studies on the prevalence of self-harm, studies investigating self-harm in children with disabilities are fewer in number and typically employ small samples. In addition, existing studies have not focused on children with wide types of disabilities, but rather focused on groups of children with specific types of disabilities like autism spectrum disorders (ASD) or ID.

Although no national data on the incidence and risk factors of self-harm in China currently exists, data from several regional studies indicate that self-harm is common in Chinese children and adolescents. Zhang et al. reported a 7.2% rate of self-injury among 2623 individuals aged from seven to twenty two years during the previous year [16]. Another study surveyed 5807 students aged 13–18 years in Beijing and found that the prevalence of self-injurious behavior during the preceding one month was 35.6% [17]. An epidemiological study of self-harm behavior among children with disabilities is scarce in China, and the risk of self-harm in Chinese children with different types of disabilities has neither been measured nor compared. Accurate estimates of the self-harm prevalence in children with different types of disabilities are needed to focus attention on the urgency and scope of this problem, allocate health resources and services, and to identify trends in self-harm over time. These knowledge gaps prompted us to study self-harm among children with disabilities. 

The aims of this study were to: (1) Examine the prevalence of self-harm in children with a variety of types of disabilities that were classified based on the China Classification and Grading Criteria of Disability; (2) compare the risk of self-harm by different types and severity of disabilities. We hypothesize that the self-harm risk differs between children with different types or severity of disabilities.

## 2. Materials and Methods 

### 2.1. Study Population 

There were 22 special education schools for disabled children distributed at 16 urban and suburban districts of Beijing, China. Of these, 11 schools were randomly selected for this study, and all agreed to participate. All children with disabilities between the ages of six and seventeen years attending the 11 schools were invited to participate. For every participating child with disabilities, we matched a child without disabilities who had the same age and gender, and who lived in the same district. Children without disabilities were recruited from general education schools.

The Institutional Review Board of Capital Medical University approved the protocol. Written consent was obtained from a parent of each child involved in our study. The ethical approved project identification code for this study is 2016SY63.

### 2.2. Disability Classification 

According to the China Classification and Grading Criteria of Disability, disability was classified into the following categories: Hearing disability, speech disability, mental retardation, physical disability, and mental health disorders; the severity of disability was grouped into four levels: Level 1 is the most severe disability level, and level 4 is the mildest degree of disability [11,18]. In addition, a disabled person was also categorized as having either a single disability or multiple disabilities. The type of disability was used to refer to both the category of disability and whether an individual had single or multiple disabilities. Individuals who had multiple disabilities were classified into one of the five categories of disability mentioned above according to the most severe type of disability he/she had. This was done in accordance with the disability type listed in his/her official certificate issued by the China Disabled Persons’ Federation (CDPF), an official agency for individuals with disability in China. Disabled children and their parents were asked to record in the questionnaire information about the type and severity of disability, which were in line with those listed in the official certificate. 

### 2.3. Health and Behavior Questionnaire

A standard questionnaire was developed, piloted and administered anonymously to children and their parents. Variables examined were those relating to demographic information (age, gender, ethnic group, parental education level, etc.), smoking, drinking, sleep problems, health and lifestyle, disabling condition, suicide ideation and behavior, and self-harm behavior. 

In this study, we hypothesized the disabling condition as a risk factor for DSH. Thus, a child must have had the disabling condition(s) for at least 12 months prior to the survey to ensure that the child’s disability was present before the act of self-harm occurred.

### 2.4. Data Collection Procedures

The collection of data took place from September 2017 until February 2018. The questionnaire was administered in classrooms in all participating schools, and took an average of about 30 min to complete. The school teacher (trained prior to the study) or a researcher in each classroom informed the children and their parents about the survey objectives and question formats. Consenting parents completed the questionnaire for their children aged 6–10 or for all disabled children who were unable to fill out the questionnaire by themselves. Children aged 11 to 17 years who were able to fill out the questionnaire completed them on their own. Parents and children were not required to provide personal identifiable information on the questionnaires. Voluntary participation, anonymity and confidentiality were assured during the whole process. Only the principal investigator had access to confidential data in these questionnaires. 

### 2.5. Self-Harm Definition 

DSH was assessed using the definition which is in line with the Child and Adolescent Self-harm in Europe (CASE) study [19].

‘A non-fatal act in which a person deliberately did one or more of the following: initiated behavior (for example self-cutting, self-burning), which they intended to cause self-harm; ingested a substance in excess of the prescribed or generally recognized therapeutic dose; ingested a recreational or illicit drug that was an act that the person regarded as self-harm; ingested a non-ingestible substance or object’. 

We used a 24-item questionnaire to classify children who engaged in DSH behaviors in the previous year into categories such as self-cutting, -hitting, taking overdoses, swallowing a non-ingestible substance, or other methods. Sample questions include, ‘have you ever deliberately cut your wrist, arms, or other part(s) of your body?’, ‘have you ever deliberately stuck sharp objects into your skin or fingers such as needles and pins?’, or ‘have you ever deliberately banged your head thereby causing a bruise?’. The response options for the 24-items were ‘no’, ‘yes, once’, ‘yes, 2–4 acts’, and ‘yes, ≥5 acts’. 

In this study, children met the study criteria for self-harm if it was reported that at least one of the acts outlined in the above 24-items had occurred, regardless of the suicidal intent. We did not judge their motives for self-harm other than to determine if the episode appeared deliberately. Determination of a child’s intent, suicidal or otherwise, during self-injury could not reliably be performed, especially for young children or those with mental retardation or mental health disorders in this study.

### 2.6. Data Analyses 

Data analyses were performed using the SAS 9.4 statistical software (SAS Institute Inc., Cary, NC, USA). We first compared the rates of DSH in children with no disability, single disability, and multiple disabilities by sociodemographic factors. We also examined the association between the severity of disability and self-harm rate by sociodemographic variables. The odds ratios (ORs) and the 95% confidence intervals (CIs) were calculated using the logistic regression analyses. We used multivariable logistic regression models to assess the self-harm risk in children who had each of five disability types, multiple disabilities, and different severity of disabilities compared with children without a disability, after controlling for potential confounding effects of the sociodemographic factors.

## 3. Results

Our study included 650 children with disabilities and 650 of their healthy counterparts. There were 406 boys and 244 girls in both of the groups. The median age of the participants was 12 years (ranging from 6–17). Due to the limited number of self-harm cases, the level 3 and level 4 severity of disability were grouped together and collectively referred to as level 3 + 4.

Table 1 shows rates of DSH that occurred during the previous year for children with no disability, single disability and multiple disabilities, stratified by sociodemographic factors. Children with a single disability or multiple disabilities had higher rates of DSH than children without disabilities (15.6% and 39.7% compared to 10.9%, *p* < 0.05). The rates of self-harm differed statistically between children with and without disabilities for the majority of sociodemographic factors. However, for children aged 14–17 years; for children whose parents were adoptive parents/stepparents; and for children with single parents, the self-harm rate did not show statistical difference between children with and without disabilities. 

In Table 2, we stratified the sampled children based on their severity of disability. Children with level 1 or level 2 disabilities had higher rates of DSH than children without disabilities (36.2% and 19.8% compared to 10.9%, *p* < 0.01). The rates of self-harm differed statistically among children with different severity of disabilities for the majority of sociodemographic factors, except the following groups: Children from ethnic minorities and children whose mother’s education was high school. Additionally, children with level 1 and level 2 disabilities had statistically higher rates of self-harm than children without disabilities throughout many of the sociodemographic categories. Notable exceptions were children who were aged 14–17, had single parents, and those whose parents had lower education levels. However, self-harm rates did not differ statistically between children in the Level 3 + 4 group and children without disabilities with the exception of children aged 6–9 years and 14–17 years.

**Figure 1:**
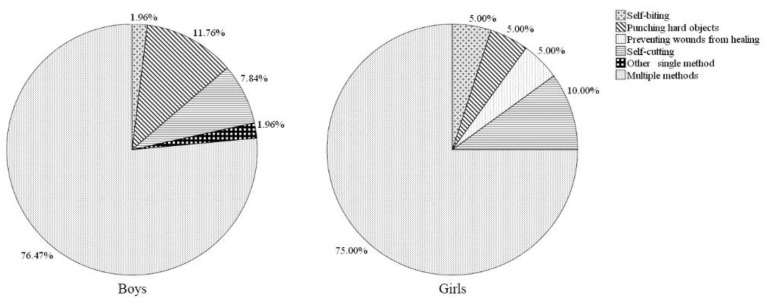
Methods of the DSH meeting study criteria during the previous year in children without disabilities, by gender.

**Figure 2:**
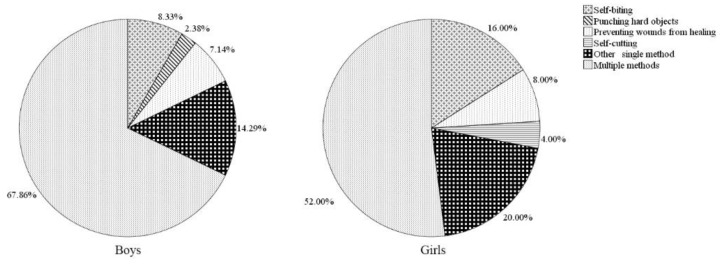
Proportion of the DSH methods in children with disabilities, by gender.

The proportions of different methods of DSH among sampled children with and without disabilities by gender are presented in Figure 1 and Figure 2. Overall, in both groups and genders, using multiple methods was the most commonly reported category of DSH. Among children without disabilities, males were more likely than females to report punching hard objects (11.8% compared to 5.0%) and using another single method (2.0% compared to 0%), but less likely to report self-cutting (7.8% compared to 10.0%), preventing wounds from healing (0 compared to 5.0%) and self-biting (2.0% compared to 5.0%). In children with disabilities, girls were more likely than boys to report using another single method (20.0% compared to 14.3%), self-biting (16.0% compared to 8.3%), and self-cutting (4.0% compared to 0%). However, none of the marked gender differences with respect to methods of self-harm in children with and without disabilities were significant (*p* > 0.05).

Table 3 shows the results of the self-harm odds ratio from univariate logistic regression models. There was a statistical difference between the minority and Han Chinese children in terms of self-harm (OR = 0.48, 95% CI = 0.29, 0.81). The self-harm rate was statistically reduced in children aged 6–9 years (OR = 0.45, 95% CI = 0.30, 0.66) and 10–13 years (OR = 0.44, 95% CI = 0.31, 0.62) compared to children aged 14–17 years.

Table 4 presents the results of the type and severity of disability on DSH among children with disabilities from multivariable logistic regression models. The reference group was children without disabilities in all logistic models. The multivariable OR of DSH among children with any disability was statistically higher than that among children without disabilities (OR = 2.40; 95% CI = 1.71, 3.36; *p* < 0.05). Children with multiple disabilities (OR = 6.89; 95% CI = 4.24, 11.20; *p* < 0.05) and level 1 severity of disability (OR = 6.11; 95% CI = 3.91, 9.56; *p* < 0.05) had the highest risk of DSH. Any single type of disability led to an increased risk of DSH (OR = 1.61, 95% CI = 1.09, 2.38). However, in regards to the specific type of disability, only children with a mental health disorder had statistically higher OR of DSH than children without disabilities (OR = 3.32; 95% CI = 2.02, 5.46, *p* < 0.05). 

## 4. Discussion

Self-harm has rarely been compared directly in children of typical development with those who have a disability [20]. In China, a study of DSH in children with different types and severity of disabilities has not previously been reported. In this study of children with disabilities aged 6–17 years and their healthy counterparts, children with disabilities had statistically higher prevalence of self-harm than children without disabilities. This study also demonstrated a clear association between the disability type, severity and DSH risk in this vulnerable Chinese pediatric population. Results reported here supported our hypothesis that the risk of self-harm differs between children with different types and severity of disabilities.

In the present study, the overall past year prevalence of DSH for children without disabilities was 10.9%, with 12.6% of males and 8.2% of females reported to engage in self-harm. This is slightly above the prevalence previously reported in an epidemiological study among children in China [16]. These results showed some differences with other countries as well. A seven-country comparative community study of DSH among adolescents around the age of 15 or 16 years reported that the past year prevalence of DSH for females ranged from 3.6% to 11.8%, with four of the seven countries reporting a rate of at least 10.4%; rates for males were between 1.7% and 4.2% [19].

Our study has shown that DSH is present in 20.6% of children with disabilities, which is statistically higher than children without disabilities. The present study supports earlier findings that many children with disabilities engage in self-harm behaviors. A meta-analysis study demonstrated that self-injury appeared to be more common amongst individuals with a severe/profound degree of ID, autism, or deficits in receptive and/or expressive communication [13]. Saloviita [21] reported a 34% prevalence of self-injurious behavior in children with mental retardation aged between zero and seventeen years. Baghdadli et al. [22] found that 50% of young children with autistic disorders experienced self-injurious behaviors. In our study, the prevalence of DSH in children with mental health disorders exceeded all of the percentages in children with other single disability types. The possible reason is that the vast majority (86.6%) of this group were children with ASD, and results from the meta-analysis indicated that individuals with a diagnosis of autism were significantly more likely to show self-injury [13].

In this study, children with multiple disabilities were at a higher risk for DSH than children with any single disability, consistent with the previous research. There is growing evidence that the prevalence of self-injury in adolescents with ASD and ID is statistically higher than that in adolescents with ID alone [23,24]. Since previous studies did not investigate self-harm in children with a broad range of types of disabilities like the present one and had different methods of data gathering, it is difficult to fully compare our results with theirs. 

Our analysis showed a strong impact of the severity of disability on the occurrence of DSH. The prevalence of DSH was 36.2% among children with level 1 (the most severe) disability, 19.8% among children with level 2 disability, and 9.2% among children with level 3 + 4 disability. These findings were in accordance with earlier reports. Baghdadli et al. [22] found that a higher degree of autism and a higher daily living skills delay were risk factors of self-injury. McTiernan et al. [25] reported that a lower IQ was associated with a higher severity and frequency of self-injury in 174 children with ASD aged 3–14. Self-harm was also found to be closely associated with the severity of mental retardation; individuals with a severe/profound level of mental retardation were significantly more likely to demonstrate self-injury than individuals with a mild/moderate level of mental retardation [13].

Previous literature has reported that DSH affects both sexes, but evidence of the association between gender and self-harm is mixed. Some studies found girls had higher prevalence rates of self-harm compared with boys [19,26,27,28], while others reported no differences between the sexes [21,29,30,31]. A study from 11 European countries found no gender differences in Hungary, Israel, Ireland, Italy and Romania with regards to occasional self-injury. In addition, there were no gender differences in terms of repetitive self-injury in Austria, Ireland, Israel and Romania, while males had statistically higher rates in Italy [29]. In this study, we found no gender differences in DSH for either children with or without disabilities. In addition, the present study did not confirm earlier findings that the method of self-harm varied statistically by gender. In contrast, we found that multiple methods were the most commonly reported method, and there were no marked gender differences according to the method of self-harm used in their most recent episode. One gender difference we found consistent with outcomes of previous research is that girls without disabilities were more likely than boys to report self-cutting only [19,29]. Boys without disabilities commonly reported punching hard objects as a method of self-harm. Both boys and girls with disabilities commonly reported another single method and self-biting. However, since none of these were statistical differences, we cannot know if these are true patterns. 

There were some limitations in this study. First, the sample size was relatively small in subgroups of children with different types of disabilities, so that some of the self-harm risk estimates in Table 4 were not stable. Our results indicated that, with the exception of mental health disorder, the odds ratio of DSH for children with individual disability types was not statistically higher than children without disabilities. This may be due to the small numbers for each of these groups and a larger study might find a statistical difference. Second, self-harm behaviors were reported retrospectively from parents or children. Earlier studies have reported that injury episodes were likely to be underestimated when using a 12-month recall period [32,33]. Third, the results of gender differences in the DSH behavior would be affected since there were much more boys in the sample than girls. Last, in the present study, we did not take into account factors like self-image, social isolation, help-rejection, limited coping skills, hopelessness, family stresses and lack of support, which might affect self-harm behaviors. Such factors could be explored in future research.

## 5. Conclusions

This study found a statistical difference in the prevalence of DSH between children with and without disabilities, and clearly demonstrated a strong impact of the type and severity of disability on the occurrence of DSH in Chinese children with disabilities. The prevalence data have added to our knowledge of the scale of the problem of self-harm, and indicate that self-harm is a significant challenge facing children with disabilities in China.

## Figures and Tables

**Table 1:** Rates of deliberate self-harm (DSH) among children with a different disability status by selected sociodemographic factors in Beijing, China.

Characteristics	Without Disabilities		Single Disability		Multiple Disabilities		*p*-Value ^a^
	*N*	Injured *n* (%)	*N*	Injured *n* (%)	*N*	Injured *n* (%)	
Total	650	71 (10.92)	514	80(15.56) *	136	54(39.71) **	<0.001
Gender							
Male	406	51 (12.56)	325	53 (16.31)	81	31 (38.27) **	<0.001
Female	244	20 (8.20)	189	27 (14.29) *	55	23 (41.82) **	<0.001
Age							
6–9	184	4 (2.17)	153	28 (18.30) **	31	14 (45.16) **	<0.001
10–13	283	20 (7.07)	210	23 (10.95)	73	27 (36.99) **	<0.001
14–17	183	47 (25.68)	151	29 (19.21)	32	13 (40.62)	0.031
Ethnic group							
Han Chinese	617	67 (10.86)	476	68 (14.29)	125	48 (38.40) **	<0.001
Ethnic minorities	33	4 (12.12)	38	12 (31.58)	11	6 (54.55) **	0.015
Parent-child relationship							
Natural parents	628	66 (10.51)	481	74 (15.38) *	128	50 (39.06) **	<0.001
Adoptive parents/stepparents	22	5 (22.73)	33	6 (18.18)	8	4 (50.00)	0.164
Single parent							
Yes	42	9 (21.43)	67	13 (19.40)	11	3 (27.27)	0.832
No	608	62 (10.20)	447	67 (14.99) *	125	51 (40.80) **	<0.001
Single child							
Yes	514	55 (10.70)	295	47 (15.93) *	75	28 (37.33) **	<0.001
No	136	16 (11.76)	219	33 (15.07)	61	26 (42.62) **	<0.001
Father’s Education							
Middle school or below	64	12 (18.75)	149	20 (13.42)	28	11 (39.29) *	0.005
High school	202	29 (14.36)	147	17 (11.56)	40	13 (32.50) *	0.004
College or above	384	30 (7.81)	218	43 (19.72) **	68	30 (44.12) **	<0.001
Mother’s Education							
Middle school or below	67	12 (17.91)	155	20 (12.90)	32	12 (37.50) *	0.004
High school	178	22 (12.36)	150	16 (10.67)	45	14 (31.11) **	0.002
College or above	405	37 (9.14)	209	44 (21.05) **	59	28 (47.46) **	<0.001
Family income per month							
<5000	99	8 (8.08)	218	31 (14.22)	55	19 (34.55) **	<0.001
5000–12,000	372	46 (12.37)	199	25 (12.56)	55	24 (43.64) **	<0.001
>12,000	179	17 (9.50)	97	24 (24.74) **	26	11 (42.31) **	<0.001

^a^*p*-values were derived from the Chi-square analysis between the disability status and self-harm prevalence, by sociodemographic factors. * and ** Children with a single disability or multiple disabilities compared with children without disabilities, respectively; ** *p* < 0.01; * *p* < 0.05.

**Table 2:** Rates of DSH among children with different severity of disabilities by selected sociodemographic factors in Beijing, China.

Characteristics	Level 1 Disability		Level 2 Disability		Level 3 + 4 Disability		*χ* ^2^	*p*-Value ^a^
	*N*	Injured *n* (%)	*N*	Injured *n* (%)	*N*	Injured *n* (%)		
Total	185	67 (36.22) **	227	45 (19.82) **	238	22 (9.24)	46.410	<0.001
Gender								
Male	124	44 (35.48) **	133	25 (18.80)	149	15 (10.07)	27.080	<0.001
Female	61	23 (37.70) **	94	20 (21.28) **	89	7 (7.87)	19.840	<0.001
Age								
6–9	43	14 (32.56) **	78	21 (26.92) **	63	7 (11.11) **	7.960	0.019
10–13	91	33 (36.26) **	92	11 (11.96)	100	6 (6.00)	33.060	<0.001
14–17	51	20 (39.22)	57	13 (22.81)	75	9 (12.00) *	12.720	0.002
Ethnic group								
Han Chinese	172	60 (34.88) **	209	37 (17.70) *	220	19 (8.64)	43.220	<0.001
Ethnic minorities	13	7 (53.85) **	18	8 (44.44) *	18	3 (16.67)	5.220	0.074
Parent-child relationship								
Natural parents	176	61 (34.66) **	208	41 (19.71) **	225	22 (9.78)	37.780	<0.001
Adoptive parents/stepparents	9	6 (66.67) *	19	4 (21.05)	13	0 (0.00)	13.030	0.001
Single parent								
Yes	18	7 (38.89)	25	7 (28.00)	35	2 (5.71)	9.290	0.010
No	167	60 (35.93) **	202	38 (18.81) **	203	20 (9.85)	38.680	<0.001
Single child								
Yes	91	33 (36.26) **	133	30 (22.56) **	146	12 (8.22)	27.950	<0.001
No	94	34 (36.17) **	94	15 (15.96)	92	10 (10.87)	20.120	<0.001
Education level of father								
Middle school or below	47	14 (29.79)	67	12 (17.91)	63	5 (7.94)	8.910	0.012
High school	62	20 (32.26) **	51	5 (9.80)	74	5 (6.76)	18.310	<0.001
College or above	76	33 (43.42) **	109	28 (25.69) **	101	12 (11.88)	22.700	<0.001
Education level of mother								
Middle school or below	56	16 (28.57)	64	11 (17.19)	67	5 (7.46)	9.580	0.008
High school	52	12 (23.08)	63	11 (17.46)	80	7 (8.75)	5.280	0.071
College or above	77	39 (50.65) **	100	23 (23.00) **	91	10 (10.99)	34.600	<0.001
Family income per month								
<5000	78	27 (34.62) **	94	17 (18.09) *	101	6 (5.94)	24.190	<0.001
5000–12,000	68	23 (33.82) **	91	14 (15.38)	95	12 (12.63)	12.820	0.002
>12,000	39	17 (43.59) **	42	14 (33.33) **	42	4 (9.52)	12.270	0.002

^a^*p*-values were derived from the Chi-square analysis of association between the severity of disability and self-harm prevalence. * Children with four levels of disability compared with children without disabilities, respectively. ** *p* < 0.01; * *p* < 0.05.

**Table 3:** Univariate logistic regression results of the DSH among children by sociodemographic factors in Beijing, China.

Variables	Sample *N*	Injured *n* (%)	Univariate Model OR (95% CI)
Gender			
Male	812	135 (16.6)	1.191 (0.87, 1.629)
Female	488	70 (14.3)	1
Age			
6–9	368	46 (12.5)	0.445 (0.301, 0.657)
10–13	566	70 (12.4)	0.439 (0.311, 0.621)
14–17	366	89 (24.32)	1
Ethnic group			
Han Chinese	1218	183 (15.0)	0.482 (0.289, 0.806)
Ethnic minorities	82	22 (26.8)	1
Parent-child relationship			
Natural parents	1237	190 (15.4)	0.581 (0.319, 1.058)
Adoptive parents/stepparents	63	15 (23.8)	1
Single parent			
Yes	120	25 (20.8)	1.462 (0.915, 2.335)
No	1180	180 (15.3)	1
Single child			
Yes	884	130 (14.7)	0.784 (0.574, 1.071)
No	416	75 (18.0)	1
Father’s education			
Middle school or below	241	43 (17.8)	1.195 (0.809, 1.767)
High school	389	59 (15.2)	0.984 (0.695, 1.393)
College or above	670	103 (15.4)	1
Mother’s education			
Middle school or below	254	44 (17.3)	1.084 (0.738, 1.592)
High school	373	52 (13.9)	0.838 (0.586, 1.199)
College or above	673	109 (16.2)	1
Family income per month			
<5000	372	58 (15.6)	0.888 (0.590, 1.337)
5000–12,000	626	95 (15.2)	0.860 (0.594, 1.245)
>12,000	302	52 (17.2)	1

**Table 4:** Multivariable logistic regression results of the type and severity of disability on DSH among children with disabilities in Beijing, China.

Characteristics	*N*	Injured *n* (%)	Univariate Model OR (95% CI) ^a^	Multivariate Model OR (95% CI) ^b^
No disability	650	71 (10.9)	1	1
Any type of disability	650	134 (20.6)	2.118 (1.551, 2.891)	2.399 (1.714, 3.358)
Any single disability	514	80 (15.6)	1.503 (1.067, 2.118)	1.608 (1.088, 2.376)
Hearing	79	11 (13.9)	1.319 (0.666, 2.612)	1.327 (0.602, 2.925)
Speech	42	0 (0.0)		
Other physical disabilities	19	1 (5.3)	0.453 (0.060, 3.445)	0.435 (0.054, 3.492)
Mental retardation	240	30 (12.5)	1.165 (0.739, 1.836)	1.190 (0.713, 1.985)
Mental health disorder	134	38 (28.4)	3.228 (2.060, 5.059)	3.317 (2.016, 5.460)
Multiple disabilities	136	54 (39.7)	5.370 (3.519, 8.196)	6.887 (4.235, 11.199)
Severity level of disability				
Level 1 (Most severe)	185	67 (36.2)	4.630 (3.141, 6.826)	6.109 (3.906, 9.555)
Level 2	227	45 (19.8)	2.016 (1.340, 3.035)	2.206 (1.391, 3.498)
Level 3 + 4	238	22 (9.24)	0.831 (0.502, 1.374)	0.817 (0.474, 1.409)

OR = odds ratio; CI = confidence interval. ^a^ ORs were calculated for each type and severity level of disability compared to no disability using the univariate logistic regression. ^b^ ORs were calculated for each type and severity level of disability compared to no disability using the multivariable logistic regression after controlling for sociodemographic factors.
